# Vertebral Compression Fractures Treated in Acute by Instrumented Kyphoplasty: Early and Mid-Term Clinical and Radiological Results

**DOI:** 10.1155/2019/1386510

**Published:** 2019-12-10

**Authors:** Jules Descamps, Mayalen Lamerain, Zied Chenguel, Perrine Jubert, Marc-Antoine Rousseau

**Affiliations:** Department of Orthopedic and Trauma Surgery, Hopital Bichat - Beaujon, Assistance Publique - Hopitaux de Paris, Paris, France

## Abstract

The treatment of traumatic low-degree vertebral compression fracture remains in a wide range between functional treatment, bracing, vertebroplasty, kyphoplasty, and even surgical fixation. The objective was to assess the innovation of instrumented kyphoplasty and to report the early and mid-term functional and radiological results. This study is a retrospective review of patients enrolled from 2012 to 2017. 104 consecutive endovertebral implantations of instrumented kyphoplasty were reviewed for the study. There were 56 women and 48 men. 93 of 104 patients were evaluated, of whom 27 were evaluated only by retrospective medical record review and 66 with follow-up visit. Clinical parameters were the pain rating scale (VAS) and the Oswestry score questionnaire. The radiological parameters were the vertebral kyphosis, vertebral height, lumbar lordosis, and adjacent disc degeneration (UCLA scale). Statistical correlations between before/after surgery/last follow-up were performed. The average follow-up was 26.7 months (3 to 55). The average VAS decreased from 8.2 to 3.2 the day after surgery, allowing immediate standup. The average Oswestry score was 14.6 at follow-up. The average vertebral kyphosis decreased from 12.9° to 6.5° post-op and stabilized at 8.0° at the last follow-up, corresponding to 28% gain on vertebral height. The lumbar lordosis was restored (+6.6°). Adjacent disc degeneration increased by 1 UCLA grade in 17 patients (16.3%) at follow-up. The instrumented kyphoplasty in acute led to immediate and lasting pain relief, with no bracing or bed rest, short stay in hospital, and quick return to daily life including professional activities. The good clinical results were associated to a stable radiological restoration of the vertebral anatomy.

## 1. Introduction

Vertebral compression fractures (VCFs) represent a major health concern. Cases can be traumatic, osteoporotic, or metastatic. Their prevalence in patients over 50 years reaches 18% in Europe and 23% in the United States [1]. The medical costs and associated economic loss are substantial; therefore, it is the 2nd most expensive fracture after the hip fracture [2]. VCF induces chronic spinal pain and leads patients to morbidity. The initial treatment remains open for debate between conservative treatment (lumbar belt/brace) and surgical treatment (vertebroplasty (VP) or kyphoplasty (KP)).

Brace is a noninvasive option. It is a treatment of choice for patients with important comorbidities with a high-risk for anesthesia and surgery. Nevertheless, tolerance to the brace had been demonstrated to be lowered in elderly patients due [3] to the period of bed rest. The average duration of conservative treatment is three months [4]. Deep vein thrombosis and pulmonary embolism, geriatric cachexia, and loss of autonomy increase the risk of mortality by 3 times [5]. In addition, a decrease in bone mineral density of 0.25 to 1% per week has also been reported in bedridden patients, increasing their bone fragility [6]. Overall, the conservative treatment has no effect on the restoration of the vertebral anatomy.

Minimal invasive surgical treatment of VCFs by cement injection under imaging guidance has gained popularity since the first use of cement injected into a tumor cervical vertebra in France in 1984 [7]. It has shown immediate and lasting effect on pain functional results and quality of life [8]. KP is a more comprehensive technique than VP since it fulfills more objectives: in addition to immediate fracture stabilization, KP intends to restore vertebral height. It has also been argued that restoring the volume of the vertebral body reduces the risk of cement leakage by reducing the pressure when the cement is introduced [9]. The first KP technique used a balloon inflation to reexpand the vertebral body before cementation. A second-generation KP uses distractible titanium implants permanently left in the vertebral body before cementation (instrumented KP). In addition to pain relief and early functional restoration, the anatomical restoration with instrumented KP may improve longer-term issues reading spinal sagittal imbalance and secondary adjacent degenerative. The objective of our study was to evaluate functional recovery and radiological restoration in VCF patients with second-generation instrumented KP, as well as to evaluate sagittal balance and potentiality of secondary adjacent discopathy.

## 2. Materials and Methods

### 2.1. Patients and Population

In our practice, patients had the choice between surgical procedure and orthopedic treatment after exposure to the advantages and disadvantages of each option.

**Figure 1:**
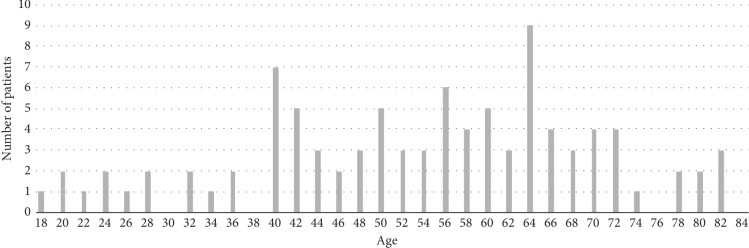
Age histogram.

**Figure 2:**
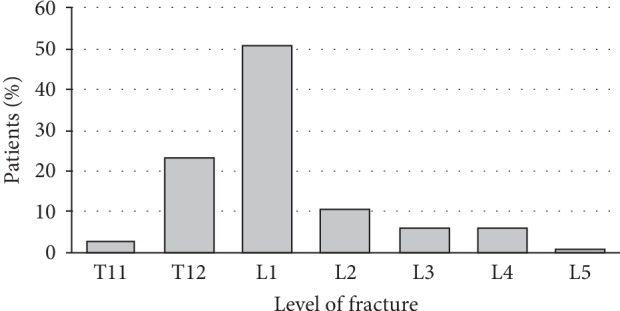
Fracture level distribution.

This study was a retrospective review of patients enrolled between May 2012 and January 2017. 104 consecutive patients with stable VCF, from the 2 hospitals in which our team worked, were all operated by instrumented kyphoplasty using SpineJack implants (Stryker) and polymethylmethacrylate cohesion cement (Stryker). These were 56 women and 48 men, with average age 54 years (18–83) with two frequency peaks around 40 and 65 years old (Figure 1). 51% of patients had a low-energy trauma, such as a fall from height. The average body mass index was 24.9 kg/m^2^. All patients had an A1 to A3 type of fracture in the Magerl classification diagnosed by a standing (if possible) X-ray anteroposterior and lateral and a preoperative CT scan [10]. Sixty-six patients have specifically been reviewed for the study; others were analyzed from their records. Two patients had died of independent causes. Only 11 patients had insufficient data and were excluded from the study (10.6%). The average follow-up was 26.7 months (3 months to 55 months). 74% of fractures involved T12 and L1 (Figure 2). The majority of the patient had Magerl A1 fractures (57.9% including 28.9% A1.3). A3 fractures were the second most frequent type (36.2% including 27.8% A3.1). The A2 represented 5.8%. The average delay between trauma and surgery was 3.5 days and 1.8 days between hospitalization and surgery. The patients were allowed to stand up without restraint at Day 0 or Day 1, except for 4 patients with associated lesions. All patients with isolated VCF went home between Day 1 and Day 3.

### 2.2. Clinical Criteria

Clinical criteria were assessed by the visual analogue scale (VAS) [11] and the Oswestry Disability Index (ODI) score [12]. The VAS was recorded preoperatively (standing position), the day after surgery and at each visit. A prefractural ODI was estimated by the patient at the time of management, and then, the score was recorded postoperatively and at each visit. All complications during or after hospitalization were collected.

### 2.3. Radiological Criteria

**Figure 3:**
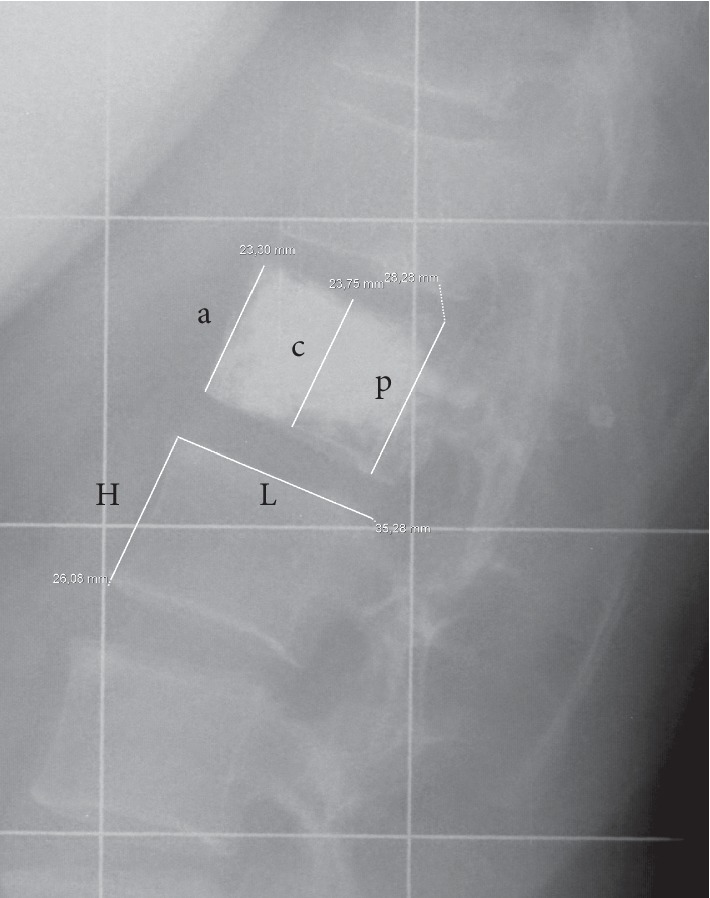
Measurement of vertebral height: a: anterior height; c: central height; p: posterior height; L: length of underlying healthy endplate; H: anterior height of the lower adjacent vertebral body.

The radiological criteria were measured at each visit on the standing images (anteroposterior and lateral views) using the Carestream Vue Pacs® Version 11.4.0.1253 software. Local parameters of interest were vertebral kyphosis (VK), regional kyphosis (RK), and regional traumatic angulation (RTA) [13]. The loss of vertebral height (anterior, central, and posterior) was measured according to the formula of Mckiernan et al. [14]. In order to standardize the data with avoiding biases due to magnification variability, the height was calibrated to the measurement of the upper endplate of the underlying vertebrae (L) (Figure 3). The regional sagittal balance was assessed by the measurement of the lumbar lordosis (LL). The relationship of theoretical lumbar lordosis and pelvic incidence (PI) was established according to the formula of Schwab et al. [15]: LL = PI ± 9 called the LL-PI mismatch. The status of the intervertebral discs was evaluated using the UCLA Grading Scale for disc degeneration in four radiographic grades [16] on each available radiograph.

### 2.4. Statistical Analysis

The statistical methodology included the use of descriptive statistics (average, standard deviation, maximum and minimum), parametric statistical tests (Fisher's test or the Wilcoxon), and nonparametric tests (Mann–Whitney). In particular, the normality of the variables was defined through the Shapiro–Wilk test of normality. Statistical significance was set at *p* < 0.05.

## 3. Results

### 3.1. Clinical Evaluation

The average VAS was 8.15 (SD = 1.74) preoperatively; 3.21 (SD = 1.52) at 24 h (*p* < 0.05 compared to preoperatively); and 1.42 (SD = 1.87) at the last follow-up (*p* < 0.05 compared to 24 h). The average prefractural ODI score was 3.8 points (SD = 7.5) preoperatively; 26.6 points (SD = 23.95) at 6 weeks (*p* < 0.05 compared to preoperatively); and then 14.6 points (SD = 16.0) at the last follow-up (*p* > 0.05 compared to preoperatively). On average, the ODI score increased by 10.2 points (SD = 14.1) between the initial status (before the fracture) and the last follow-up. At last follow-up (mean 26.7 months and median 27 months), 53 patients (70.6%) had an ODI below 20 points. The return to daily activity was obtained between 7 and 10 days postoperatively, and the return to professional activity was obtained between 10 days and 7 months (average of 60 days). An adjacent vertebra fracture occurred in a 63-year-old osteoporotic woman who fell from her height 3 months after surgery.

### 3.2. Radiological Assessment

**Figure 4:**
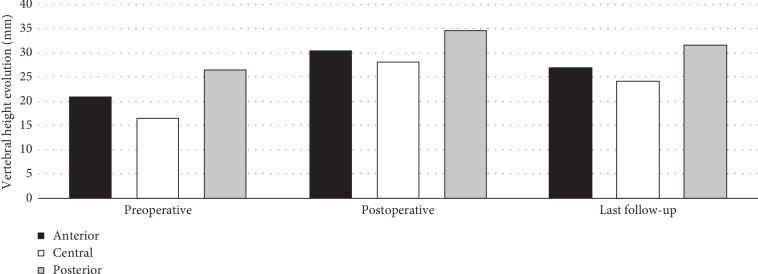
Evolution of vertebral height (mm).

VK, RK, and RTA changes are reported in Table 1. The height gain was greater at the central part with an increase of 71% at 24 h compared to its preoperative height and 48% at the last follow-up. For the anterior part, the height increase was 46% after the operation and 29% at the last follow-up. The posterior wall increase was 12% after the surgery and 3% at the last follow-up (Figure 4).

The average pelvic incidence was 53.4° (SD = 12.2). The average lumbar lordosis was 42.1° (SD = 9.7) preoperatively; 46.4° (SD = 9.2) at 24 h; and 48.7° (SD = 10.9) at the last follow-up. The variation of the lordosis and LL-PI mismatch are reported in Table 2.

Three patients (3.3%) had grade 1 preoperative disc degeneration at the superior adjacent level. No disc degeneration of the inferior adjacent disc was diagnosed preoperatively. At the last follow-up (mean 29 months), 17 patients had increased their degeneration disc by 1 grade. At the last follow-up, 20.3% had superior adjacent disc disease radiological signs. No degeneration sign of the inferior adjacent disc was observed (Table 3).

**Figure 5:**
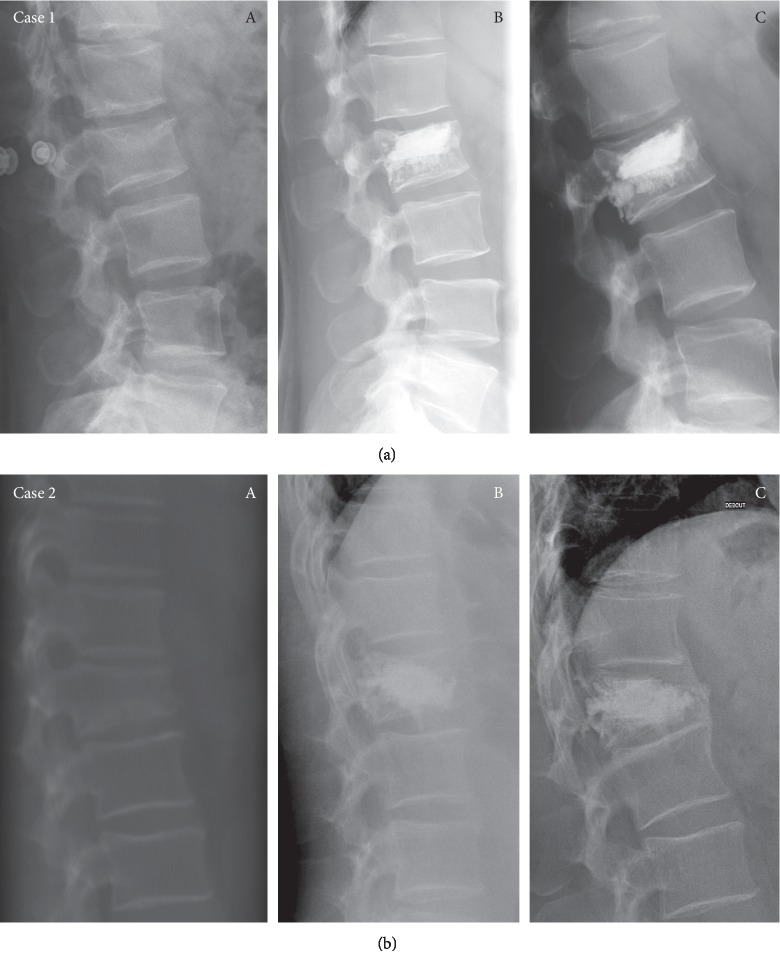
Examples of good and worst cases. (a) Case 1: M42y L2: preoperative (A), postoperative (B), last follow-up at 32 months (C). (b) Case 2: M63y T12: preoperative (A), postoperative (B), last follow-up at 35 months (C).

In total, no neurological or postoperative embolic complications have been recorded. 12 patients (11.6%) had cement leaks with no clinical consequences. 58.3% of the leaks were lateral.  Figure 5 illustrates an excellent and a worst case.

## 4. Discussion

Our study reported good immediate clinical results and stable anatomical restoration over time in terms of local and regional radiological parameters. In spite of sagittal balance restoration, some extent of degenerative evolution of adjacent disc was noted, which did not impair the clinical results.

The clinical results showed significant and immediate efficacy on the VAS within the first 24 h post-op. The only other study published using the SpineJack (Stryker) implants reported better immediate VAS improvement [17] at 48 h post-op time point. Overall, the ODI results at last follow-up were superimposable with those of Noriega et al. [17] with an average of 14.6 points at follow-up in our study and a median of 4.4 points for Noriega et al. (the median was 8 points in our study). The gain of ODI is not strictly comparable since the preoperative status is commonly a postfractural status (or unspecified), while we decided to use an estimated preoperative prefractural status based on the patients' declaration.

Our series reported an improvement of the VK from 12.9° to 6.5° in the same range compared to the 14.5° to 9.2° change in the series of Noriega et al. [17] with the same surgical device for instrumented kyphoplasty. In comparison, Ateş et al. [18] reported a change for the VK from 33° to 17° using noninstrumented balloon kyphoplasty. Patients in their study had much severe initial kyphosis, and their VCF might not be strictly comparable to ours. We presented additional results with using the calculation of the RTA to our report. We notice that changes in RK were not significant while changes in RTA were significant, probably because RTA limits biases related to the level. This shall help for better comparisons with other studies coming.

The restoration of vertebral height is another way to assess the quality of the reduction. We observed 46% immediate gain in anterior height in our series between the pre-op and post-op status. There were no data to refer to in the literature for instrumented KP. The study of Ates et al. regarding noninstrumented KP reported 11.8% improvement [18]. VP and conservative treatment did not allow vertebral height restoration [19]. To have a better understanding, we sought to study separately the three vertebral heights, anterior, central, and posterior. To us, the instrumented KP allows effective restoration on the central part of the vertebra. We did not notice any recollapse of cemented vertebra although we had a population with risk factors [20, 21]. In our worst case shown in Figure 5, no. 2 had an important loss of reduction at the last FU. We could explain this evolution first of all; with regard to implant positioning, the implant could have been more anterior in the vertebra. The anterosuperior part of the vertebra, forming a triangle, is free of implants or cement, making it a weaker area. Also, it is the bone quality of this patient with a thin vertebral cortex; too much stress could cause a wedge-shaped deformity of the body of the vertebra.

Kyphosis and vertebral height are involved in the forward sagittal imbalance, which is significantly associated with pain and loss of function [22]. The improvement of the lumbar lordosis by 6.6° in average in our series shall be compared to VCF series reporting the same outcome. Using noninstrumented KP, Yokoyama et al. reported 2.9° increase in lordosis and sagittal vertical axis (SVA) decreased [23]. We also presented our data in terms of LL-PI mismatch after the work of Schwab et al. [15]. We notice that changes in the lumbar lordosis were not statistically significant while changes in LL-PI mismatch were significant, probably because taking into account the variability of the individual sagittal balance helps reading the results. We think these additional data would limit biases and help further comparisons.

Assessment of stability of the radiological restoration in a longer term was another goal of our study. The secondary loss of VK improvement in our series was 1.5° between post-op and last follow-up (6.55° to 8.04°). In comparison, Hiwatashi et al. [19] reported that the vertebral height was 17.2 mm after surgery to 16.4 mm at final follow-up using VP treatment (i.e., maintained but not corrected). The recent study of Andrei et al. [24] suggests that the measurement of the increase of vertebral volume might be even more significant. In the study of Hartmann et al. [25], VK passed from 8.7° (DS = 6.93) initially to 5.8° (DS = 5.97) postoperatively and finally 8.1° (DS = 6.09) at the last follow-up using the balloon KP, showing return to the initial status. We obtain from our study that the instrumented KP made it possible to achieve some degree of reduction that was maintained at follow-up.

Regarding the progression of the disc degeneration, we observed in our series a mild evolution in the upper adjacent disc with 16.3% of cases worsening by 1 grade at follow-up on plain radiographs. Benneker et al. found that the radiological analysis is as reliable as MRI analysis with a strong correlation for the assessment of disc degeneration [26]. In a series of 97 patients (62 VP or KP/35 conservative) aged 65.3 years in average and followed over 3.9 years, Qian et al. [27] reported that 52.6% of cases evolved to disc degeneration after VP or noninstrumented KP (respectively, 66.7% and 45.3%) vs 29% after conservative treatment. In comparison, the occurrence of secondary disc degeneration in our series seems less than that after conservative treatment. We hypothesize that the global restoration of the sagittal balance and the local restoration of the endplate may account for it, as suggested by Teyssédou et al. who previously concluded that the correction of vertebral plate deformation avoided the loss of mechanical function in the disc [28]. However, 16.3% remains a significant rate. Despite the study of Loriaut et al. [29] who suggested that the initial trauma did not clearly affect the disc in the initial phase, Su et al. [30] demonstrated a correlation between the severity of the fracture and the disc disease. It is likely that the disruption of the endplate has double consequence: mechanical and biological since it plays a role in the nutrition of the adjacent disc [31]. König et al. reported a recurring 1.3% disc disease at 15 months with KP [32], as did Verlaan et al., for whom the discs adjacent to the VCF did not seem to evolve towards severe degeneration after more than a year of follow-up after pedicle screw fixation and direct endplate reduction with KP [33]. For us, the potential role of initial trauma, the actual biological properties of the healed endplate, the amount of restoration of its concave shape, and the final sagittal balance in a global standpoint are the beginning of understanding the natural history of the adjacent disc degeneration in the context of VCF.

This study has several limitations: having a noncomparative plan, the comparison is made only with the medical literature. It was retrospective and without a control group; hence, the evidence regarding the effectiveness of the surgery rather than the natural evolution of the healing process is limited. Nor did we consider any difference in disc degeneration between the lumbar and thoracic discs. Also, we did not distinguish subgroups of patients with high-energy and low-energy trauma which may implicate osteoporosis fracture.

In conclusion, the instrumented kyphoplasty in acute provides immediate and lasting pain relief, with no bracing or bed rest and short stay in hospital. The good clinical results were associated to a stable radiological restoration of the vertebral anatomy.

## Figures and Tables

**Table 1:** Evolution of the angles of vertebral kyphosis (VK), regional kyphosis (RK), and regional traumatic angle (RTA): preoperative, postoperative, and at last follow-up.

	Preoperative		Postoperative		Last follow-up	
	Mean	SD	Mean	SD	Mean	SD
Vertebral kyphosis (°)	12.9	5.7	6.5^*∗*^	4.8	8.0^*∗*^^,^^*∗∗*^	6.1
Regional kyphosis (°)	11.4	6.3	10.2	8.8	10.4	6.4
Traumatic regional angulation (°)	4.4	7.5	2.7^*∗*^	6.5	3.2^*∗*^	7.3

^*∗*^Significant change compared to the preoperative time. ^*∗∗*^Significant change compared to the postoperative time.

**Table 2:** Variation in lumbar lordosis (LL) and difference between LL and pelvic incidence (PI): preoperative, postoperative, and at last follow-up.

	Preoperative		Postoperative		Last follow-up	
	Mean	SD	Mean	SD	Mean	SD
Pelvic incidence (°)	54.4	12.2				
Lumbar lordosis (°)	42.1	13.8	46.4	10.9	48.7	14.5
PI—LL (°)	11.5	9.7	5.7^*∗*^	9.2	5.8^*∗*^	10.9

^*∗*^Significant change compared to the preoperative time.

**Table 3:** Evolution the grade of disc degeneration (UCLA scale): preoperative and last follow-up.

UCLA grading scale	Preoperative				Last follow-up			
	I	II	III	IV	I	II	III	IV
Overlying disc (%)	96.7	3.3	0.0	0.0	79.8	17.9	2.4	0.0
Underlying disc (%)	100.0	0.0	0.0	0.0	95.2	3.6	1.2	0.0

## Data Availability

The data used to support the findings of this study are available from the corresponding author upon request.
